# Accelerating FLAIR imaging via deep learning reconstruction: potential for evaluating white matter hyperintensities

**DOI:** 10.1007/s11604-024-01666-5

**Published:** 2024-09-24

**Authors:** Noriko Nishioka, Yukie Shimizu, Yukio Kaneko, Toru Shirai, Atsuro Suzuki, Tomoki Amemiya, Hisaaki Ochi, Yoshitaka Bito, Masahiro Takizawa, Yohei Ikebe, Hiroyuki Kameda, Taisuke Harada, Noriyuki Fujima, Kohsuke Kudo

**Affiliations:** 1 Department of Diagnostic and Interventional Radiology, Hokkaido University Hospital, Sapporo, Japan; 2 Department of Diagnostic Imaging, Faculty of Medicine and Graduate School of Medicine, Hokkaido University, Sapporo, Japan; 3 Medical Systems Research & Development Center, FUJIFILM Corporation, Tokyo, Japan; 4 FUJIFILM Healthcare Corporation, Tokyo, Japan; 5 Center for Cause of Death Investigation, Faculty of Medicine, Hokkaido University, Sapporo, Japan; 6 Faculty of Dental Medicine, Department of Radiology, Hokkaido University, Sapporo, Japan; 7 Division of Medical AI Education and Research, Hokkaido University Graduate School of Medicine, Sapporo, Japan

**Keywords:** FLAIR, Deep learning reconstruction, White matter hyperintensity, Accelerated image

## Abstract

**Purpose:**

To evaluate deep learning-reconstructed (DLR)–fluid-attenuated inversion recovery (FLAIR) images generated from undersampled data, compare them with fully sampled and rapidly acquired FLAIR images, and assess their potential for white matter hyperintensity evaluation.

**Materials and methods:**

We examined 30 patients with white matter hyperintensities, obtaining fully sampled FLAIR images (standard FLAIR, std-FLAIR). We created accelerated FLAIR (acc-FLAIR) images using one-third of the fully sampled data and applied deep learning to generate DLR–FLAIR images. Three neuroradiologists assessed the quality (amount of noise and gray/white matter contrast) in all three image types. The reproducibility of hyperintensities was evaluated by comparing a subset of 100 hyperintensities in acc-FLAIR and DLR–FLAIR images with those in the std-FLAIR images. Quantitatively, similarities and errors of the entire image and the focused regions on white matter hyperintensities in acc-FLAIR and DLR–FLAIR images were measured against std-FLAIR images using structural similarity index measure (SSIM), regional SSIM, normalized root mean square error (NRMSE), and regional NRMSE values.

**Results:**

All three neuroradiologists evaluated DLR–FLAIR as having significantly less noise and higher image quality scores compared with std-FLAIR and acc-FLAIR (*p* < 0.001). All three neuroradiologists assigned significantly higher frontal lobe gray/white matter visibility scores for DLR–FLAIR than for acc-FLAIR (*p* < 0.001); two neuroradiologists attributed significantly higher scores for DLR–FLAIR than for std-FLAIR (*p* < 0.05). Regarding white matter hyperintensities, all three neuroradiologists significantly preferred DLR–FLAIR (*p* < 0.0001). DLR–FLAIR exhibited higher similarity to std-FLAIR in terms of visibility of the hyperintensities, with 97% of the hyperintensities rated as nearly identical or equivalent. Quantitatively, DLR–FLAIR demonstrated significantly higher SSIM and regional SSIM values than acc-FLAIR, with significantly lower NRMSE and regional NRMSE values (*p* < 0.0001).

**Conclusions:**

DLR–FLAIR can reduce scan time and generate images of similar quality to std-FLAIR in patients with white matter hyperintensities. Therefore, DLR–FLAIR may serve as an effective method in traditional magnetic resonance imaging protocols.

## Introduction

Magnetic resonance imaging (MRI) is widely used in medical diagnostics and currently serves as the gold standard for investigating most central nervous system disorders. Owing to its exceptional ability to suppress cerebrospinal fluid signals, fluid-attenuated inversion recovery (FLAIR) imaging is considered one of the most valuable imaging sequences for white matter hyperintensity evaluation, and thus is routinely employed in clinical practice [1, 2].

However, FLAIR imaging, which employs an inversion recovery technique for water signal suppression, inherently results in a lower signal-to-noise ratio, thereby lengthening acquisition time and rendering the sequence more susceptible to subject motion; this may increase noise and artifacts, potentially compromising diagnostic accuracy [3]. Recent advancements in rapid imaging techniques have highlighted their clinical benefits, including reduced scan time and enhanced image quality. Notably, shorter examination time is preferred to maximize patient compliance. Various acceleration techniques have been developed to address the limitations of traditional FLAIR imaging. Parallel imaging (PI) methods, such as sensitivity encoding, offer the advantage of faster image acquisition using phased-array coils to facilitate image reconstruction with fewer data points [4, 5]. Similarly, compressed sensing has emerged as a promising approach for accelerating MRI acquisition by exploiting sparsity in image representations and employing iterative reconstruction algorithms [6–8]. Despite these advancements, challenges remain, including signal-to-noise ratio losses associated with high acceleration factors (AFs) in PI techniques and the computational intensity of iterative compressed sensing algorithms, especially for high-resolution images [9, 10].

To address these challenges, various deep learning-based MRI reconstruction methods have been developed with the expectation of enhancing image quality and reducing scan time [11]. Recently, deep learning-based reconstruction methods have demonstrated rapid development across various fields of radiology [12–15], including brain MRI [16, 17], with a growing focus on adapting FLAIR sequence [18–21]. With the progress in research in this area, it is important to verify the high quality of deep learning-generated images and evaluate their clinical applicability. In particular, for FLAIR images, which are used in white matter hyperintensity assessment, it is necessary to validate whether images generated by deep learning accurately depict both normal structures and white matter hyperintensities. However, studies on the verification of deep learning-reconstructed FLAIR (DLR–FLAIR) images for accurately rendering both normal structures and white matter hyperintensities are limited.

The aim of this study was to evaluate whether DLR–FLAIR images obtained rapidly using undersampled data can substitute fully sampled images in clinical settings. By focusing on images and white matter hyperintensities, we qualitatively and quantitatively compared three types of images: fully sampled FLAIR, rapidly acquired FLAIR, and rapidly acquired DLR–FLAIR images.

## Materials and methods

Our institutional review board approved this retrospective, single-center study, and the need for written informed consent was waived owing to the retrospective nature of the study.

### Patient data

Patient data were collected exclusively at our institution. The study cohort comprised individuals who met the following criteria: (a) previously underwent FLAIR MRI revealing multiple white matter hyperintensities, (b) underwent follow-up MRI between July 2022 and October 2022, (c) were imaged using a specific MRI device capable of storing raw data used for this investigation, and (d) had successful acquisition of raw FLAIR data.

### MRI image acquisition

All patients were scanned using a 3-T MRI scanner with a 32-channel head coil (FUJIFILM Healthcare Corporation, Tokyo, Japan).

The acquisition parameters for two-dimensional (2D) axial FLAIR were as follows: repetition time 12,000 ms, echo time 118.8 ms, inversion time 2770 ms, flip angle 90 $^\circ$, field of view 240 × 240 mm, reconstructed matrix 512 × 512 (acquired matrix 320 × 376), slice thickness 5 mm, and slice gap 1.5 mm. The scan time of fully sampled data was 4 min 37 s. The image obtained using this fully sampled method was referred to as the standard FLAIR (std-FLAIR) image.

We retrospectively reconstructed 3 $\times$ accelerated images (i.e., images obtained by applying AF = 3) using one-third of the fully sampled raw k-space data. MATLAB (version 2022a, MathWorks Inc., Natick, MA, USA) was used for the reconstruction. Intel Core i9-7900X CPU (Intel Inc., CA, USA) was used, with a memory of 32 GB. Images with a 3 $\times$ AF were generated by first performing a Fourier transform on the fully sampled complex image of each channel to generate a k-space signal. This signal was then reduced by performing equidistant under-sampling in the phase encoding direction, sampling every third line. The 3 $\times$ accelerated image was generated from the reduced k-space signal using a conventional reconstruction method (sensitivity encoding) or iterative reconstruction. An image equivalent to that obtained via 3 $\times$ acceleration was designated as an accelerated FLAIR (acc-FLAIR) image.

To estimate the anticipated scan time for 3 $\times$ acc-FLAIR, the time required for full sampling was divided by the AF (AF = 3).

### Deep learning-based reconstruction

PI produces spatially non-uniform noise when the AF is high. To reduce non-uniform noise, we developed a method that combines iterative reconstruction with soft-thresholding and deep-learning-based denoising, with details published in the literature [22]. Our iterative reconstruction method was based on projections onto convex sets (POCS) [23]. In the POCS method, noise amplification depended on the sensitivity distribution, and spatial noise non-uniformity was generated. Wavelet-based soft-thresholding was added to the POCS method to reduce noise amplification. After spatial noise nonuniformity was reduced via wavelet-based soft-thresholding, spatial noise uniformity was reduced via deep learning-based denoising. Deep learning-based denoising combines a super-resolution convolutional neural network [24] with a residual network [25]. Super-resolution convolutional neural network comprises three layers, and each layer performs convolution, bias, and rectified linear unit operations. The kernel size was 9 × 9 for the first layer and 5 × 5 for the second and third layers. The first, second, and third layers had 64, 32, and 1 channels, respectively. Training was performed using reference image/noisy image pairs [22].

We applied deep learning-based reconstruction to the 3 $\times$ acc-FLAIR images and designated the resulting images as DLR–FLAIR images.

### Qualitative analysis (reader study)

#### Image quality evaluation

As part of the qualitative assessment, three board-certified neuroradiologists with 14, 10, and 9 years of experience in radiology, respectively, visually evaluated the std-FLAIR, acc-FLAIR, and DLR–FLAIR images of all patients. All FLAIR images were anonymized and randomly displayed. The images were independently and blindly evaluated by the three neuroradiologists using a dedicated viewer (VOX-BASE Browser/View; J-Mac System, Sapporo, Japan).

To assess the image quality, two aspects were emphasized: (i) the amount of noise and (ii) the visibility of the gray/white matter contrast in the frontal lobes. Each aspect was rated on a 4-point scale. The amount of noise was evaluated as follows: the image contained excessive noise, and thus was unsuitable for diagnosis (1 point); noise was present, potentially impacting the diagnostic interpretation (2 points); noise was minimal, posing little to no effect on diagnosis (3 points); and almost no noise was evident, providing minimal diagnostic limitations (4 points). The visibility of gray/white matter contrast in the frontal lobes was assessed as follows: obscure (1 point), partially obscure (2 points), mostly visible (3 points), and fully visible (4 points).

#### White matter hyperintensity identification

Two radiologists with 15 and 17 years of experience, distinct from the three evaluators, selected 100 white matter hyperintensities, each measuring 3–10 mm in diameter. These hyperintensities were identified from the std-FLAIR images of 30 patients. Each patient had at least one hyperintensity, with a preference for those situated at the level of the centrum semiovale. The 100 hyperintensities were randomly displayed using combinations of std-FLAIR and either acc-FLAIR or DLR–FLAIR images. The same three board-certified neuroradiologists who assessed the image quality evaluated hyperintensity visibility in acc-FLAIR and DLR–FLAIR images compared with that in std-FLAIR images.

The boundaries of hyperintensities were rated on a 4-point scale as follows: unclear and different from std-FLAIR (1 point), partially different (2 points), nearly identical (3 points), and equivalent (4 points).

### Quantitative analysis

#### Image quality

The structural similarity index measure (SSIM) and normalized root mean square error (NRMSE) were calculated as pixel-based quantitative measures across the full image set, encompassing the entire brain. SSIM values (ranging from 0 to 1) indicate the percentage deviation of the acc-FLAIR and DLR–FLAIR images from the std-FLAIR image, with higher values denoting greater similarity to std-FLAIR image [26]. The NRMSE, a normalized variant of the root mean square error, offers a scale where lower values suggest less error, and thus closer resemblance between the acc-FLAIR or DLR–FLAIR and the std-FLAIR baseline image.

#### White matter hyperintensity identification

In addition, regions of 25 × 25 pixels containing 100 extracted white matter hyperintensities were harvested from each image at precisely the same coordinate axis. For the 100 white matter hyperintensities, the SSIM and NRMSE values in the region of 25 × 25 pixels (regional SSIM and regional NRMSE) for acc-FLAIR and DLR–FLAIR images were computed and compared with those for the std-FLAIR image.

MATLAB (The MathWorks Inc.) was used for the quantitative analysis.

### Statistical analysis

The neuroradiologist-assigned image quality assessment scores for std-FLAIR, acc-FLAIR, and DLR–FLAIR were evaluated for statistical significance using the Wilcoxon signed-rank test. Bonferroni-corrected *p* values were employed, with statistical significance defined as a *p* value < 0.05. Results are discussed based on these corrected *p* values. The neuroradiologist-assigned white matter hyperintensity identification and quantitative (SSIM, NRMSE, regional SSIM, and regional NRMSE) scores for acc-FLAIR and DLR–FLAIR images were evaluated for statistical significance using the Wilcoxon signed-rank test. Statistical significance was defined as a *p* value < 0.05.

For qualitative assessment, the intraclass correlation coefficient (ICC) (2,1) was employed to evaluate inter-rater reliability among the evaluators (neuroradiologists) with values falling into the following categories: < 0.50 (poor), 0.50–0.75 (moderate), 0.75–0.90 (good), > 0.90 (excellent) [27].

The Wilcoxon signed-rank test was performed using JMP Software (version 16.1.0; SAS Institute Inc., Cary, NC, USA), and the ICC (2,1) was calculated using the Bell Curve for Excel (Social Survey Research Information Co., Tokyo).

## Results

All *p* values reported in this study have been Bonferroni-corrected.

### Patient data

**Fig. 1:**
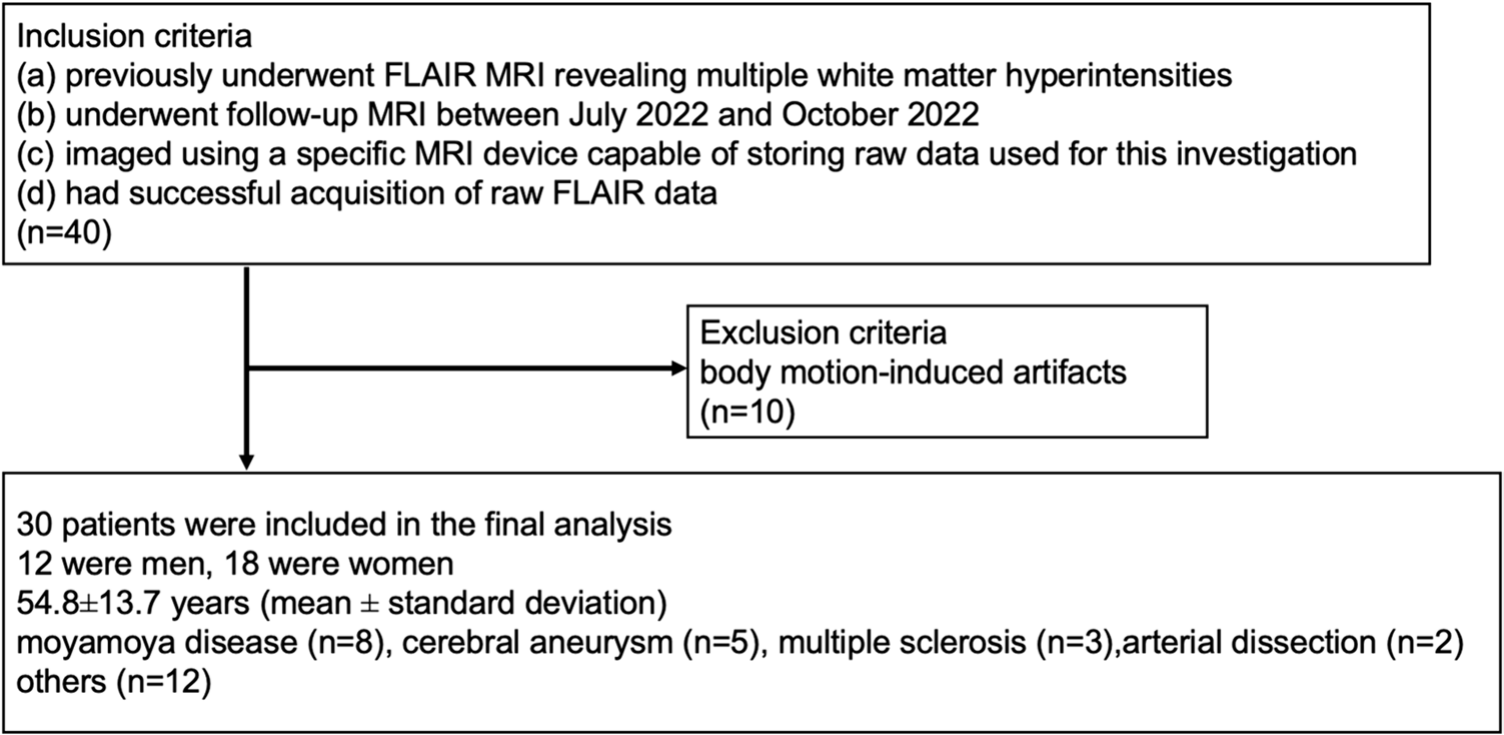
Flowchart of patient inclusion and exclusion

A flowchart of patient inclusion and exclusion is provided in Fig. 1. Forty patients met the inclusion criteria. However, 10 patients were excluded because of body motion-induced artifacts. Subsequently, data from 30 patients were included in the final analysis. Of the 30 patients, 12 were men and 18 were women. The age of the study cohort was 54.8 ± 13.7 years (mean ± standard deviation).

MRI was performed in the 30 patients for the following conditions: moyamoya disease (*n* = 8), cerebral aneurysm (*n* = 5), multiple sclerosis (*n* = 3), arterial dissection (*n* = 2), and one case each of white matter lesions, acquired immunodeficiency syndrome, postoperative treatment of vascular malformation, hypertension, headache, post-internal carotid artery stent placement, brainstem lesion, cavernous sinus syndrome, chronic pain, hypothalamic lesion, cerebellar arteriovenous malformation, and treatment after vestibular schwannoma.

The 100 white matter hyperintensities selected for this study included those reflecting the pathologies of the aforementioned conditions, as well as ischemic changes and old infarcts, which are considered to be less directly related to the primary underlying pathologies.

### Estimated image acquisition time

The calculated scan times for acc-FLAIR and DLR–FLAIR were approximately 1 min and 32 s, respectively.

### Qualitative analysis (reader study)

**Fig. 2:**
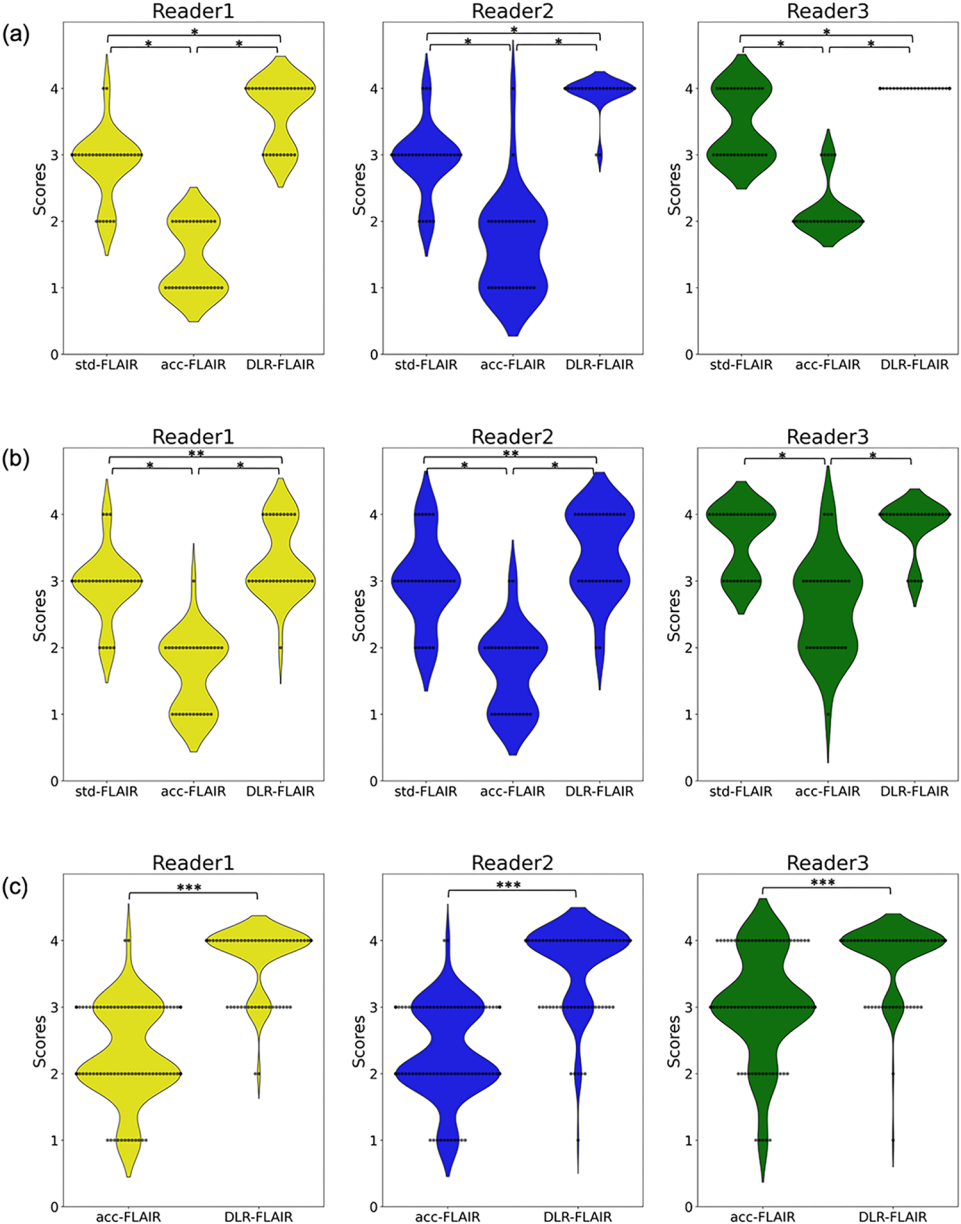
Scores assigned by the three neuroradiologists for each image type. **a** Amount of noise. **b** Visibility of gray/white matter contrast in the frontal lobes. **c** White matter hyperintensity identification. *p < 0.001, **p < 0.05, ***p < 0.0001. *FLAIR* fluid-attenuated inversion recovery, *acc* accelerated, *DLR* deep learning-reconstructed, *std* standard

**Fig. 3:**
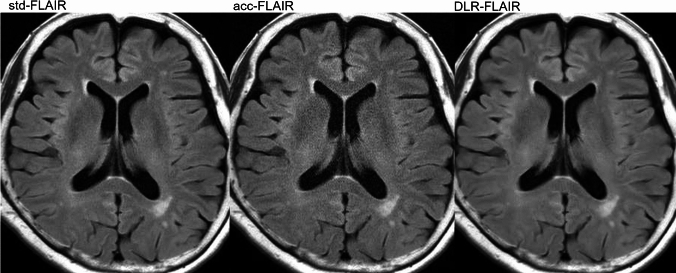
Entire representative FLAIR images from three reconstruction methods

**Fig. 4:**
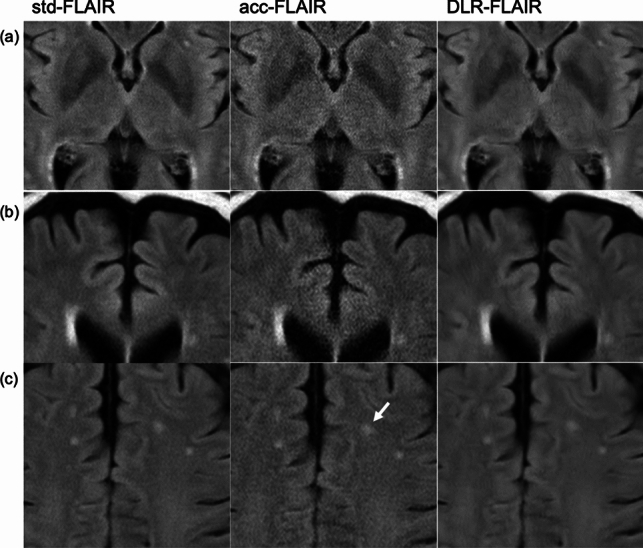
Enlarged and partially cropped sections of three reconstruction methods. **a** In acc-FLAIR imaging, there is a noticeable amount of noise in the central area, resulting in an overall grainy image. In contrast, std-FLAIR and DLR–FLAIR imaging have less noise, with DLR–FLAIR displaying less noise compared with std-FLAIR. **b** In acc-FLAIR imaging, the central area shows excessive noise, which obscures the gray/white matter contrast in certain areas. Conversely, DLR–FLAIR imaging, by removing noise, achieves a gray/white matter contrast comparable to that of std-FLAIR imaging. **c** In acc-FLAIR imaging, the white matter hyperintensity appears slightly larger due to the noise blurring its edge (arrow). DLR–FLAIR imaging shows hyperintensity edges that are equivalent to those in std-FLAIR imaging. *FLAIR* fluid-attenuated inversion recovery, *acc* accelerated, *DLR* deep learning-reconstructed, *std* standard

Figure 2 shows the image quality an hyperintensity identification scores assigned by the three neuroradiologists to std-FLAIR, acc-FLAIR, and DLR–FLAIR. Figure 3 presents the entire representative FLAIR images from these three reconstruction methods. Figure 4 displays an enlarged and partially cropped section of the same three reconstruction methods.

Regarding image quality based on noise level evaluation, the scores assigned by the three neuroradiologists were as follows (mean ± standard deviation): Reader 1 assigned scores of 2.87 ± 0.50, 1.43 ± 0.50, and 3.67 ± 0.47; Reader 2 assigned scores of 2.93 ± 0.51, 1.63 ± 0.71, and 3.93 ± 0.25; Reader 3 assigned scores of 3.47 ± 0.50, 2.17 ± 0.37, and 4.00 ± 0.00, for std-FLAIR, acc-FLAIR, and DLR–FLAIR, respectively. All three neuroradiologists significantly favored std-FLAIR and DLR–FLAIR over acc-FLAIR (*p* < 0.001). Moreover, they assigned significantly higher scores for DLR–FLAIR than for std-FLAIR (*p* < 0.001).

Concerning the image quality based on the assessment of the visibility of gray/white matter contrast in the frontal lobe, the scores assigned were as follows (mean ± standard deviation): Reader 1 assigned scores of 2.93 ± 0.51, 1.63 ± 0.55, and 3.30 ± 0.53; Reader 2 assigned scores of 3.00 ± 0.63, 1.67 ± 0.60, and 3.43 ± 0.62; Reader 3 assigned scores of 3.63 ± 0.48, 2.63 ± 0.71, and 3.83 ± 0.37, for std-FLAIR, acc-FLAIR, and DLR–FLAIR, respectively. All three neuroradiologists assigned significantly higher scores for std-FLAIR and DLR–FLAIR than for acc-FLAIR (*p* < 0.001). In the comparison between std-FLAIR and DLR–FLAIR, two neuroradiologists (Reader 1 and Reader 2) assigned significantly higher scores for DLR–FLAIR (*p* < 0.05). One neuroradiologist (Reader 3) tended to assign higher scores for DLR–FLAIR images, although the difference from other scores was not statistically significant (*p* = 0.0942).

**Fig. 5:**
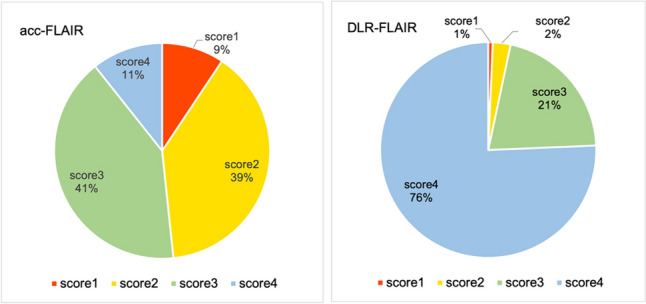
Distribution of neuroradiologist scores for white matter hyperintensities in acc-FLAIR and DLR–FLAIR images relative to those in std-FLAIR images. In acc-FLAIR imaging, neuroradiologists attributed 1 or 2 points for 48% of the white matter hyperintensities. This suggests that nearly half of the hyperintensities in acc-FLAIR images differed in appearance from those in std-FLAIR images. In contrast, in DLR–FLAIR imaging, neuroradiologists assigned 3 or 4 points for 97% of the hyperintensities, implying that the hyperintensity depiction in DLR–FLAIR imaging is comparable to that seen in std-FLAIR. FLAIR, fluid-attenuated inversion recovery; acc, accelerated; DLR, deep learning-reconstructed; std, standard

**Fig. 6:**
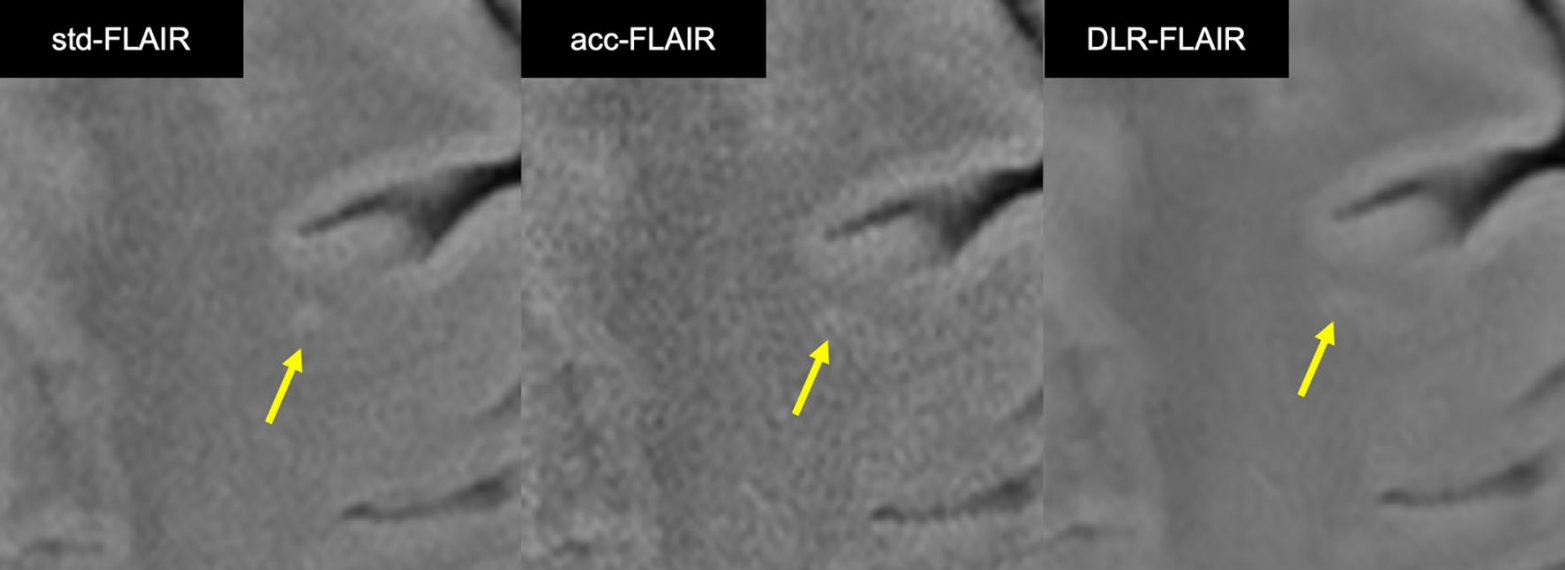
Hyperintensity that appeared differently in acc-FLAIR and DLR–FLAIR compared to std-FLAIR. In the acc-FLAIR image, the hyperintensity shows more noise and appears differently in shape compared to std-FLAIR image. In the DLR–FLAIR image, while the noise is removed, the hyperintensity shape still differs from that in the std-FLAIR image. This hyperintensity was scored 1 by all neuroradiologists for acc-FLAIR and by two neuroradiologists for DLR–FLAIR

Concerning white matter hyperintensity evaluation, hyperintensity appearance resemblances of acc-FLAIR and DLR–FLAIR images were compared with those of std-FLAIR images. The scores assigned by the three neuroradiologists for acc-FLAIR vs. DLR–FLAIR images were as follows (mean ± standard deviation): Readers 1, 2, and 3 assigned scores of 2.27 ± 0.69 vs. 3.76 ± 0.47, 2.30 ± 0.69 vs. 3.63 ± 0.63, and 3.02 ± 0.80 vs. 3.76 ± 0.51, respectively. All three neuroradiologists significantly favored DLR–FLAIR images by assigning higher scores (*p* < 0.0001 for all comparisons), indicating that the hyperintensities depicted by DLR–FLAIR were more similar to those depicted by std-FLAIR. The distribution of the scores assigned by the three neuroradiologists for the 100 white matter hyperintensities is shown in Fig. 5. Notably, 52% of the hyperintensities depicted by acc-FLAIR images and 97% of those depicted by DLR–FLAIR images were rated 3 (nearly identical to std-FLAIR images) and 4 (equivalent to std-FLAIR images) points, respectively. Figure 6 shows a hyperintensity that appeared differently in acc-FLAIR and DLR–FLAIR compared to std-FLAIR. This hyperintensity was rated as "hyperintensity boundary visibility was different from that in std-FLAIR and unclear (1 point)" by all evaluators for acc-FLAIR, and it was scored 1 point by both Reader 2 and Reader 3 for DLR–FLAIR. The ICC (2,1) for inter-rater reliability is summarized in Table 1. Although the inter-rater reliability was good between Readers 1 and 2 (0.776–0.798), it was poor to moderate between Readers 1 and 3 as well as between Readers 2 and 3 (0.462–0.761).

**Table 1:** Values of the intraclass correlation coefficient (ICC) (2,1) for inter-rater reliability

		ICC (2,1)	95% confidence interval	
	Reader		Lower limit	Upper limit
Noise	Reader1-2	0.798	0.702	0.864
	Reader1-3	0.699	0.162	0.869
	Reader2-3	0.761	0.495	0.873
Visibility	Reader1-2	0.778	0.682	0.848
	Reader1-3	0.462	– 0.033	0.725
	Reader2-3	0.514	0.045	0.747
White matter hyperintensity	Reader1-2	0.776	0.715	0.826
	Reader1-3	0.527	0.345	0.657
	Reader2-3	0.621	0.338	0.769

### Quantitative analysis

A quantitative comparison of the overall image quality of acc-FLAIR and DLR–FLAIR image qualities against that of std-FLAIR as a benchmark showed significantly higher SSIM values for DLR–FLAIR images (0.964 ± 0.0077) than for acc-FLAIR images (0.919 ± 0.0136), suggesting a closer resemblance between DLR–FLAIR and std-FLAIR images. Furthermore, DLR–FLAIR images exhibited a significantly lower NRMSE value (0.0131 ± 0.0018) than acc-FLAIR images (0.0178 ± 0.0026), indicating reduced deviation from std-FLAIR images. Regarding white matter hyperintensity analysis, DLR–FLAIR images showed significantly higher regional SSIM (0.99992 ± 0.00003) and significantly lower regional NRMSE values (0.0637 ± 0.0180) than acc-FLAIR images (0.99978 ± 0.00009 and 0.0948 ± 0.0292, respectively) (Table 2).

**Table 2:** Results of quantitative analysis

	SSIM	NRMSE
acc-FLAIR	0.919 ± 0.0136*	0.0178 ± 0.0026*
DLR–FLAIR	0.964 ± 0.0077*	0.0131 ± 0.0018*

	Regional SSIM	Regional NRMSE
acc-FLAIR	0.99978 ± 0.00009*	0.0948 ± 0.0292*
DLR–FLAIR	0.99992 ± 0.00003*	0.0637 ± 0.0180*

(expressed as mean ± standard deviation)

^*^p < 0.0001

## Discussion

In this study, we qualitatively and quantitatively evaluated three types of images: std-FLAIR, acc-FLAIR, and DLR–FLAIR. In the qualitative assessment, DLR–FLAIR images exhibited the lowest noise levels and best visibility of gray/white matter contrast in the frontal lobe. Conversely, acc-FLAIR images demonstrated the highest noise levels and poorest contrast visibility. Furthermore, 97% of white matter hyperintensities in DLR–FLAIR images were rated 3 (nearly identical to std-FLAIR images) or 4 (equivalent to std-FLAIR images) points, indicating that DLR–FLAIR closely matches std-FLAIR in white matter hyperintensity depiction. In the quantitative evaluation, DLR–FLAIR showed significantly higher SSIM and lower NRMSE values compared to acc-FLAIR. However, regional SSIM values for white matter hyperintensity areas were similarly high for both DLR–FLAIR and acc-FLAIR, despite the clear visual differences. This suggests that SSIM values may not always align with human perception. Overall, this study demonstrates that deep learning-based reconstruction technology significantly improves the visual quality of FLAIR images obtained from undersampled data. This advancement can potentially be applied in clinical settings, enhancing the efficiency and quality of FLAIR imaging.

The efficacy of deep learning-based noise reduction in brain MRI has been previously reported. Kidoh et al. demonstrated that applying a denoising approach with DLR–FLAIR sequences imaged with a reduced number of image acquisitions can yield an image quality equivalent to or better than that obtained with a higher number of image acquisitions, while also reducing the scan time [18]. Tajima et al. reported that by adapting a denoising approach with deep learning reconstruction technology to 1.5-T brain MRI images, including FLAIR sequences, they achieved an image quality nearly equivalent to or surpassing that of 3.0-T brain images [19]. However, these studies were conducted on healthy individuals, and thus the image quality was not examined in terms of hyperintensity visibility. Yamamoto et al. reported potential image quality improvements, lesion identification enhancement, and scan time diminution after the application of noise reduction via deep learning-based reconstruction to accelerated three-dimensional (3D) FLAIR MRI images of patients with multiple sclerosis [21]. Estler et al. demonstrated that accelerated DLR–FLAIR sequences (scan time 1 min 39 s) improved image quality and diagnostic reliability while reducing examination time by 38% compared with std-FLAIR sequences (scan time 2 min 40 s) in the assessment of brain metastases [20]. In this study, we did not focus on specific diseases but rather on white matter hyperintensities present in patients with diverse underlying conditions. The DLR–FLAIR images generated from undersampled data depict these hyperintensities similar to those in std-FLAIR images. This suggests that DLR–FLAIR has the potential to accelerate FLAIR imaging and be widely adopted in clinical practice. Yamamoto et al. and Estler et al. applied deep learning reconstruction to data obtained through prospective k-space undersampling. In contrast, our study employed retrospective reconstruction. Prospective undersampling reduces the data during the imaging process itself, while retrospective undersampling applies this reduction after full data acquisition. There are distinct differences between prospective and retrospective undersampling, especially in subjects with minor to severe motion or low SNR. While we excluded cases with motion artifacts, some differences compared to studies using prospective undersampling might still arise.

Our study has a few limitations. First, the small sample size of 30 patients underscores the need for future research with larger sample sizes to validate our findings, as the limited number may not fully represent the diverse array of clinical presentations. Second, our analysis was limited to 2D-FLAIR imaging, and thus did not explore the potential of artificial intelligence (AI)-based denoising techniques in other MRI sequences (such as T2- and T1-weighted imaging) or 3D imaging modalities (such as 3D-FLAIR imaging). Further research should extend AI-based denoising application to a broader sequence spectrum to evaluate its overall utility and effectiveness. Third, the under-sampling rate in this study was limited to 3 × AF. Future studies should consider applying deep learning reconstruction techniques to images with higher under-sampling rates to determine their effectiveness with even less data. Furthermore, various sampling patterns should also be considered in future research to assess their potential benefits. Fourth, our study evaluated only the overall image quality and the visibility of white matter hyperintensities, without assessing the detection capability of these hyperintensities. Future research should apply deep learning reconstruction to images with higher under-sampling rates and evaluate its effectiveness in detecting white matter hyperintensities. Finally, the AI-based denoising method employed in our study was specific to MRI systems from a single manufacturer, raising questions regarding its transferability to devices from other manufacturers. Future studies should assess the performance of this technique across various MRI platforms to ensure its adaptability and wider clinical applicability.

In conclusion, DLR–FLAIR has the potential to reduce acquisition time and generate images of similar quality to std-FLAIR in patients with white matter hyperintensities. These findings suggest that DLR–FLAIR could be widely applied in routine clinical practice.
